# Oral Absorption across Organotypic Culture Models of the Human Buccal Epithelium after E-cigarette Aerosol Exposure

**DOI:** 10.1021/acsomega.2c06304

**Published:** 2022-12-01

**Authors:** Masato Miyauchi, Shinkichi Ishikawa, Takeshi Kurachi, Kazutami Sakamoto, Hideki Sakai

**Affiliations:** ∥ Tobacco Science Research Center, R&D Group, Japan Tobacco Inc., 6-2 Umegaoka, Aoba, Yokohama, Kanagawa 227-8512, Japan; ⊥ Scientific Product Assessment Center, R&D Group, Japan Tobacco Inc., 6-2 Umegaoka, Aoba, Yokohama, Kanagawa 227-8512, Japan; § Department of Pure and Applied Chemistry, Faculty of Science and Technology, Tokyo University of Science, 2641 Yamazaki, Noda, Chiba 278-8510, Japan

## Abstract

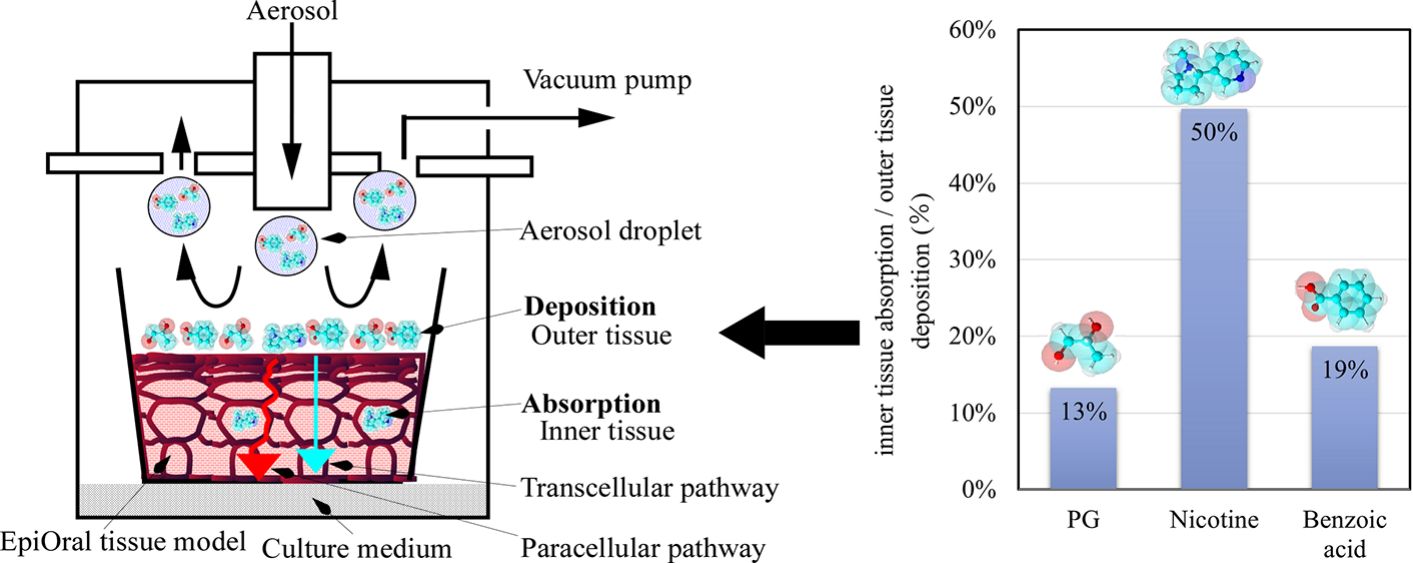

Inhaled aerosols are absorbed across the oral cavity, respiratory tract, and gastrointestinal tract. The absorption across the oral cavity, which is one of the exposure routes, plays an important role in understanding pharmacokinetics and physiological effects. After aerosol exposure from e-cigarettes, tissue viability studies, morphological observation, and chemical analyses at the inner and outer buccal tissues were performed using organotypic 3D in vitro culture models of the buccal epithelium to better understand the deposition and absorption on the inner and outer buccal tissues. The aerosol exposures did not affect the tissue viability and had no change to the tissue morphology and structure. The deposition ratio at the buccal tissue surface is relatively low. This shows that majority of aerosol transfers to the airway tissues. The distribution from the inner tissue to the outer tissue has selectivity among various compounds, depending on the affinity with the liquid crystal structure of phospholipids and glucosylceramide. Although nicotine absorption in the aqueous solution was well known to increase as the unprotonated state of nicotine increased, the nicotine absorption after the aerosol exposure is irrelevant to the protonated–unprotonated state. Furthermore, the results showed that half of nicotine that adhered to the oral cavity transferred to the inner tissue via the oral epithelium and the other half transferred to the gastrointestinal tract accompanying multiple executions of swallowing, while majority of the water-soluble compounds with the hydroxyl group such as propylene glycol and benzoic acid that adhered to the oral cavity were eluted with the saliva and transferred to the gastrointestinal tract by swallowing.

## 1 Introduction

When aerosols are exposed to the oral cavity, aerosols adhere to the buccal tissue and are transported to the body’s blood and tissues via the buccal epithelium. At this time, the variation of concentrations in the body’s blood and tissues, which is referred as the pharmacokinetic profile, has been estimated by the absorption across the oral cavity, respiratory tract, and gastrointestinal (GI) tract, liver metabolism, and urine excretion.^1^ The pharmacokinetic profile plays an important role in assessing the biological effects of various tobacco products such as cigarettes, smokeless tobacco, and electronic cigarettes.^1,2^ The deposition ratio of the particle/vapor phase in the oral cavity, bronchi, and lungs after cigarette smoking has been published.^3^ The deposition of particles or gas/vapor across the bronchi and alveoli reaches 39.7% or 45.5% of the inhaled smoke, respectively.^3^ As a result, the concentrations of nicotine in plasma rapidly reached the maximum value immediately after cigarette smoking via the transpulmonary route.^4^ It has been noted that the aerosol released from an e-cigarette effectively transfers to the airway tissue.^4^ However, the deposition of particles or gas/vapor across the oral cavity reaches 22.6% or 44.4% of the inhaled smoke, respectively.^3^ The deposition level per surface area onto the oral epithelium is also higher than that onto the epithelium of any other organ.^3^ Therefore, we have focused on the relationship between the oral epithelium and e-liquid aerosol absorption.

The oral cavity tissues are entirely covered by a stratifying squamous epithelium. A non-keratinizing epithelium such as buccal epithelium occupies approximately 60% of the oral cavity area, while a keratinizing epithelium such as gingiva occupies approximately 25% of the area.^5,6^ The rest is the dorsum of the tongue. The keratinizing epithelium consists of the stratum basal, stratum spinosum, stratum granulosum, and stratum corneum, while the non-keratinized epithelium consists of the stratum basal, stratum spinosum, intermediate stratum, and superficial stratum.^5^ The stratum corneum in the keratinizing epithelium with a thickness of 15–30 μm and the intermediate and superficial stratum in the non-keratinizing epithelium with a thickness of 250–400 μm act as an absorption barrier across the epithelium.^7^ The keratinized regions have an absorption less than that of the non-keratinized regions.^5−7^

As cells leave the basal layer and enter into differentiation, they begin to accumulate keratin and lipids, and a part of the accumulating lipid is packaged in small organelles known as “membrane-coating granules (MCGs)” or “lamellar granules”.^5^ The nutrients or waste products are delivered to or removed from the epithelium by diffusion between the epithelium and the capillary beds in the underlying connective tissue.^5^ In the final stages of the differentiation process, the MCGs migrate to the apical end of the epithelium and the lamellar lipid contents are extruded into the paracellular spaces.^6^ The time taken by a cell to divide and pass through the entire epithelium is called the turnover time. It is estimated to be 14 days in the oral epithelium, as compared to approximately 28 days in the epidermis.^8,9^

An increase in the distribution of the MCGs including the β-glucosidase activity and glucosylceramides or ceramides was observed in the direction of the apical end of the epithelium and to be in the conversion of glucosylceramides to ceramides in the final differentiation stage of the keratinized epithelium.^5,7,10^ The distribution of the MCGs, which form the neutral lipid (ceramide) sheets in the paracellular region, has been reported to support a relationship between the MCGs and the absorption of various epithelia.^5^ The regional variation of the lipids of the epidermis, gingival epithelium, palatal epithelium, and buccal tissues and floor of the mouth may contribute to a difference in the barrier function.^6,11−13^

The epithelium has tight junctions connecting cells at the upper end of the stratum granulosum, which function as a biodefense that prevents the entry of harmful components such as pathogens. However, for the buccal epithelium, it has been reported that the barrier is not based on tight junctions.^14^ The following two models have been proposed for the epithelium barrier formation. First, Norlén presented the model by which the MCG lamellae are released extracellularly to form a multilamellar lipid matrix of the stratum corneum paracellular region.^15^ The membrane-coating granule fractions, derived from the Golgi complex, have a high lipid content, which consists of sphingomyelin, phosphoglycerides, cholesterol, glucosylceramides, ceramides, and several other neutral lipids, and a number of acid hydrolases, which consist of glucosidase, sphingomyelinase, and phospholipases. The skin surface is coated with lipids secreted by the sebaceous glands. This lipids consist of squalene, wax esters, triglycerides, fatty acids, cholesterol esters, and cholesterol.^5,11^ In the final differentiation stage of the keratinized epithelium, MCG-containing glucosidase leads to deglycosylation from glucosylceramide to ceramides, forming lipid lamellar structures of ceramide, cholesterol, and fatty acids.^5,11^ Then, their fractions are transformed into the paracellular lamellar structure by a membrane fusion process.^5,11^ On the other hand, in the non-keratinized epithelium, its intermediate MCG stays in the cell and remains in the lamellar structure of glucosylceramide.^5,11^ There are still some unsolved questions with the Norlén model: (1) energy cost, (2) lack of membrane continuity, and (3) time cost, where it is thermodynamically disadvantageous, being accompanied with changes in the curvature and the like. Alternatively, the membrane folding model was proposed where epithelium barrier morphogenesis may take place via a continuous and highly dynamic process of intersection-free membrane unfolding with a concomitant crystallization of the emerging multilamellar lipid structure, which is related to the curvature energy.^15^ That is, the paracellular lipids undergo a phase transition from a bicontinuous cubic phase to a lamellar structure. The transition from a bicontinuous cubic to a lamellar lipid phase involves flattening of the folded bilayer while keeping the high mobility (i.e., liquid crystalline state) of the constituent lipids, and the lipid structure is dehydrated and deglycosylated at the interface between the stratum granulosum and stratum corneum.^15^

The MCG lamellae of the non-keratinized epithelium are considered to be of the bicontinuous cubic phase as an intermediate. The evidence to enhance keratinization of the epithelium via dehydration is also consistent with the model.^15,16^ Additionally, the absorption mechanism in the keratinized epithelium is divided into paracellular and transcellular pathways, and the absorption is thought to be characteristic of diffusion across a lipid phase. The keratinized stratum corneum cells are known to be dominated by the permeation of the paracellular pathway.^5,6^ The paracellular lipids in the keratinized stratum corneum are well-known to be of a gel state close to hydrated crystals, which consist mainly of ceramide, fatty acids, and cholesterol, and are arranged in two unit cells with repeat distances of a long-period lamellar structure (13.6 nm) of hexagons (0.42 nm) and a short-period lamellar structure (6 nm) of rectangles (0.42 and 0.37 nm) or a domain structure of a liquid crystal.^17,18^

Oleic acid, l-menthol, and d-limonene are believed to promote the absorption across the keratinizing epithelium by being accompanied with an efflux of cholesterol from the lamellar structure of ceramide–fatty acid–cholesterol and a decrease in the periodicity of the paracellular lipid lamellar structure including the liquid crystal lamella.^19^ On the other hand, in the non-keratinizing epithelium, only approximately 5–6% glucosylceramide has been converted to ceramide,^12^ and this suggests that the high absorption onto the non-keratinizing epithelium cells was dominated by the absorption across the phospholipid membranes or the lipid structure at the intermediate stratum.

Recently, organotypic 3D in vitro culture models of the buccal epithelium have been used to study the absorption, irritation, oral care, and toxicity of the oral cavity.^19−23^ To better understand the absorption onto the non-keratinizing epithelium cells, we performed tissue viability, scanning electron microscopy (SEM) observation of morphological changes, and chemical analysis after e-cigarette aerosol exposure by using the organotypic culture models of the human buccal epithelium. Furthermore, we discussed the absorption mechanism from the viewpoint of the selectivity and the effect of oral absorption on the human exposure route.

## 2 Experimental Section

### 2.1 Organotypic Tissue Culture Models

EpiOral tissue, which represents a highly differentiated, three-dimensional, cultured buccal tissue equivalent, was purchased from MatTek Corporation (Ashland, MA). The buccal tissue (EpiOral, ORL-200-PC6.5, transwell inserts of 6.5 mm in diameter on a polycarbonate membrane) is of 8–12 cell layers and approximately 50 μm in thickness and non-cornified.^19,20,23^ The lipid content of the EpiOral tissue is similar to that of the native buccal tissue; only low levels of ceramide 2 (Ceramide NS) are present. Additionally, glucosylceramide and cholesterol are present, but the lipid ratio of phospholipids to glucosylceramides of the EpiOral tissue was about 1/20 less than that of the native buccal tissue.^12,20,24−28^ Before the aerosol exposure, the inserts were separated from the agarose of the shipping plate, transferred to a plate containing 700 μL of medium, and then equilibrated at 37 °C in 5% CO_2_ for more than 1 h.

### 2.2 Aerosol Generation and Exposure

**Figure 1:**
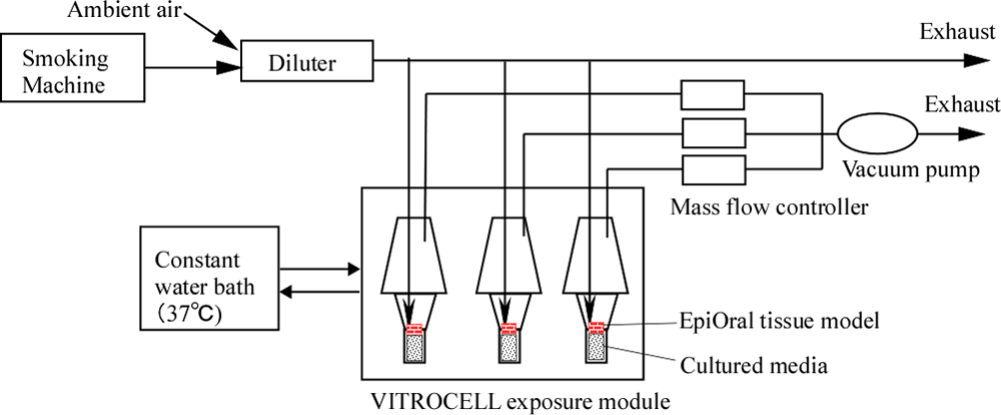
Schematic representation of aerosol generation and exposure module.

Various types of aerosol exposure systems have been developed and applied to in vitro testing.^29^ The Vitrocell exposure system used in this study has been reported to be used for in vitro aerosol exposure experiments.^30−32^ A commercially available e-cigarette was obtained from eDNC2 (Electronic Direct Heating Nicotine System Platform 2 Generation 0 version b), and aerosol was generated from e-liquids A and B. Acid free e-liquid A is composed of propylene glycol (PG) and glycerin (G) mixtures in a PG:G mass ratio of 3:7 and 4 wt % nicotine. E-liquid B is composed of the same content of PG, G, and nicotine as e-liquid A and, additionally, 5 wt % benzoic acid. PG, G, nicotine, and benzoic acid were purchased from FUJIFILM Wako Pure Chemical Corporation (Osaka, Japan). Considering the fraction of protonated and unprotonated nicotine,^33^ the aerosol of e-liquid B had a higher ratio of the protonated nicotine in the aerosol compared to acid-free e-liquid A, and the impact of nicotine absorption properties on the EpiOral tissue can be investigated. E-cigarettes were puffed for approximately 34 min, equating to 100 puffs, which is defined as a 55 mL puff drawn over 3 s with 20 s intervals and using 3.6 s exhaust, by using a smoking machine (RGA-System R26, Borgwaldt Technik, Germany). The EpiOral tissue was directly exposed at the air–liquid interface to the diluted mainstream aerosol in a Vitrocell 12/3 glass module within a climatic chamber (VITROCELL Systems GmbH, Waldkirch, Germany) at 37 °C, which is shown in Figure 1.^32^ Usually, the exposure test was performed by placing the EpiOral tissue within three chambers of the exposure module. In an absorption study, one of the chambers was placed with an insert (no tissues) and filled with only 320 μL of water to determine the value of the aerosol exposure.^32^ Five milliliters of air with the aerosol per minute is constantly introduced per chamber of the exposure module by a mass controller, and aerosols are exposed to the EpiOral tissue. The outlet gas is open to a fume hood.

The exposure tests were as follows. After e-liquid A or B was loaded into the eDNC2 cartridges, aerosols generated from eDNC2 were directly exposed to the EpiOral tissue. Upon exposure, the EpiOral tissue was placed in the exposure module to provide the culture medium (MatTek Corporation, ORAL-200-MM) and was warmed to 37 °C by a water jacket.

### 2.3 Methyl Thiazolyl Tetrazolium (MTT) Assay

The tissue viability after the aerosol exposure was determined using the MTT assay, which is well known to be an effective quantitative means to predict skin irritation.^20^ MTT assay tests were performed in triplicate for the positive and negative control, air-exposure group, and aerosol exposure with e-liquids A and B. The negative control was the untreated tissue. For the positive control, 40 μL of 1% Triton X-100 were applied to the apical surface of the tissue and tissues were then incubated at 37 °C, 5% CO_2_, for 1 h.

The MTT assay test was initiated simultaneously for each sample and was performed in the order of the negative control, air exposure, e-liquid A exposure, e-liquid B exposure, and positive control. The MTT assay protocol and the calculation of viability was described by a previous report.^20,27,28,34^ After the exposure, the tissues were rinsed with PBS, loaded with a 600 μL solution of MTT reagent (MatTek Corporation, MTT-100) in a culture medium for 3 h (37 °C), blotted to remove unreacted MTT, and then extracted in 1.1 mL of isopropyl alcohol (FUJIFILM Wako Pure Chemical Corporation) by shaking for 2 h at room temperature. Next, 200 μL of the extracted solution was pipetted into a 96-well assay plate, and the optical density (OD) was determined at 550 nm using a plate reader (SpectraFluor, Tecan, Switzerland). The OD of MTT extract at 550 nm was measured as a blank. Percent viability was calculated from the ratio of the ODs for the difference between the samples and blank. All data obtained were analyzed using GraphPad Prism software version 9.3.1 (GRAPH PAD software Inc., California, USA). One-way ANOVA followed by Dunnett’s post hoc test against the negative control was performed to determine significant differences between samples and negative controls when the value of *P* < 0.05 was considered statistically significant.

### 2.4 Scanning Electron Microscopy (SEM) Observation

As mentioned above, the non-keratinizing epithelium enhances the keratinization of epithelium via the dehydration. To confirm that no keratinization occurred during the aerosol exposure, SEM observations of the EpiOral tissue after the aerosol exposure were performed. After fixation in glutaraldehyde (FUJIFILM Wako Pure Chemical Corporation), the specimens were rinsed in 0.2 M phosphate buffer and post-fixed with 1% osmium tetroxide (FUJIFILM Wako Pure Chemical Corporation). After rinsing, the specimens were dehydrated in ethanol (FUJIFILM Wako Pure Chemical Corporation) and embedded in propylene oxide (Nisshin EM Co. Ltd., Tokyo, Japan) and epoxy resin (TAAB Laboratories Equipment Ltd., Berks, England). Thereafter, the tissues were cut into the cross-sectional specimens by a microtome (Ultracuts, Leica Microsystems, Wetzlar, Germany). The tissues were determined by the SEM (JSM-7800F, JEOL, Tokyo, Japan) at an accelerating voltage of 5 kV.

### 2.5 Tissue Absorption Studies

To understand barrier properties in the buccal tissue, the distribution of the inner EpiOral tissue absorption and outer EpiOral tissue deposition of e-liquid compounds was quantified. Since quantitation of glycerin might be affected by the glycerin derivates present in the EpiOral tissue and cultured medium, PG, nicotine, and benzoic acid, except glycerin, after the aerosol exposure were extracted in water and analyzed.

#### 2.5.1 Quantitation of Values of the Outer EpiOral Tissue

To analyze the e-liquid value of the outer EpiOral tissue, the EpiOral tissue after the aerosol exposure was separated from the transwell insert and immersed into an Eppendorf tube containing 300 μL of water. Thereafter, the procedure of removing the cells and freshly immersing them in 300 μL of water and recovering them was repeated two times. The sequential three extracts were mixed to serve as extracts of e-liquid of the outer EpiOral tissue (approximately 900 μL was collected). To confirm the validity of the extract amount, spike and recovery tests were performed using nicotine, benzoic acid, and propylene glycol aqueous solution at the given concentration instead of water.

#### 2.5.2 Quantitation of Values of the Inner EpiOral Tissue

To extract the e-liquid value from the inner EpiOral tissue of the residual tissue in Section 2.5.1, the residual EpiOral tissue was crushed with a homogenizer (BioMasherIII, Nippi Incorporated, Tokyo) to dispense 100 μL of water, and the supernatant was obtained by centrifugation. Thereafter, the procedure of dispensing 100 μL of water in the sediment and crushing then centrifuging was repeated two times. The sequential three supernatants were mixed to make 300 μL, and we added another 300 μL of water (approximately 600 μL was collected) to serve as a sufficient extract of e-liquid of the inner EpiOral tissue. For the extract of the inner tissue, spike and recovery tests were performed in the same manner as with the extract of the outer tissue.

#### 2.5.3 Determination of E-liquid Amounts during the Aerosol Exposure

The e-liquid amounts of aerosol exposure into the EpiOral tissue were determined by collecting and quantifying solutions from inserts filled with only 320 μL of water after the aerosol exposure. The reason why water was applied as a trap is that aerosol adheres to the buccal epithelial surface based on the physical collision, and the buccal epithelium is covered with the aqueous solution (i.e., saliva) and their solubilities in various artificial saliva and water are almost the same.

#### 2.5.4 Quantitation of Nicotine, Benzoic Acid, and PG

The quantitation of nicotine and benzoic acid was performed by liquid chromatography (ACQUITY, Waters, Massachusetts, USA) coupled to a photodiode array detector (PDA) and a mass spectrometer (Synapt G2-S, waters, Massachusetts, USA). Separation was achieved using a Unison UK-C18UP (IMTAKT, 100 mm × 2 mm i.d., i.d. 3 μm). The mobile phase eluents were 10 mM ammonium acetate buffer and acetonitrile with a flow rate of 0.4 mL/min and a temperature of 40 °C. The elution initial composition was maintained for 1 min at a 100% initial mobile phase, changing by linear gradient to 100% acetonitrile over the course of 6 min and holding constant for 8 min. The eluents were returned to the original condition of the 100% initial mobile phase to allow re-equilibration of the system. The nicotine and benzoic acid were detected by 260 nm of PDA and the negative ion mass spectra at *m*/*z* 121, respectively. The quantitation of PG was performing by gas chromatography (GC-2010, Shimazu, Kyoto Japan) coupled to a flame ionization detector after diluting the aliquot sample twofold with ethanol. Separation was achieved using a DB-WAXETR (Agilent J&W, 30 m × 0.53 mm i.d., i.d. 2.0 μm). The carrier gas was helium with a flow rate of 12 mL/min. The column temperature was maintained at 130 °C for 0.5 min, rising to 140 °C at a rate of 20 °C/min, and then to 180 °C at a rate of 40 °C/min and held constant for 0.5 min, and finally to 240 °C at a rate of 50 °C/min and held for 2.3 min.

All data obtained were analyzed using Excel for Microsoft 365 for Welch’s t-test and GraphPad Prism software version 9.3.1 (GRAPH PAD software Inc., California, USA) for Tukey’s multiple comparison procedure. Welch’s t-test for the comparison of liquid-A exposure and one-way ANOVA followed by Tukey’s post hoc test for the comparison of liquid B was performed to determine significant differences between samples when the value of *P* < 0.05 was considered statistically significant.

## 3 Results and Discussion

### 3.1 MTT Assay Test

**Figure 2:**
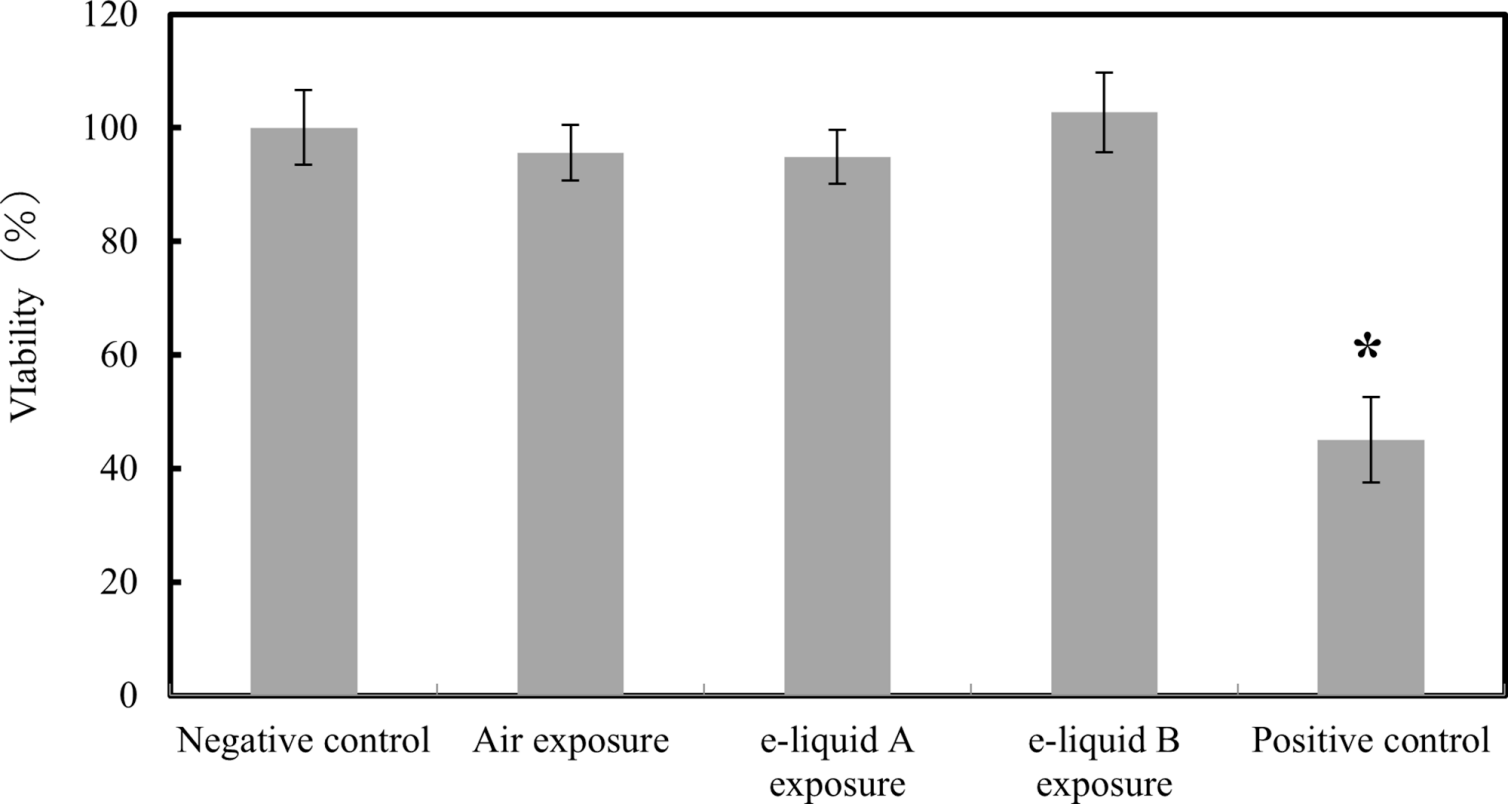
Effect of exposure on EpiOral tissue viability. *P < 0.05 (one-way ANOVA followed by Dunnett’s post hoc test against the negative control).

The MTT assay depends on the reduction of MTT (tetrazolium salt methyl thiazoyl tetrazolium) by mitochondrial dehydrogenases.^20^ The main mitochondrial activity in the epithelium occurs in the basal layers. Thus, we can detect any damage on the basal cell layers when the exposed compounds permeate into the epithelium.^35,36^ The viabilities of the EpiOral tissue are shown in Figure 2. Data obtained showed a viability of 95–103% by any aerosol e-liquid exposure, as compared with the positive control of 45%, and no effect of the e-liquid aerosol exposure on the tissue viability. In addition, the viability of the air exposed was 96%. All exposure groups used in this study did not affect the viability. Therefore, the e-cigarette aerosol showed no decrease in the tissue viability, which has been published by Neilson.^28^

### 3.2 SEM Observation

**Figure 3:**
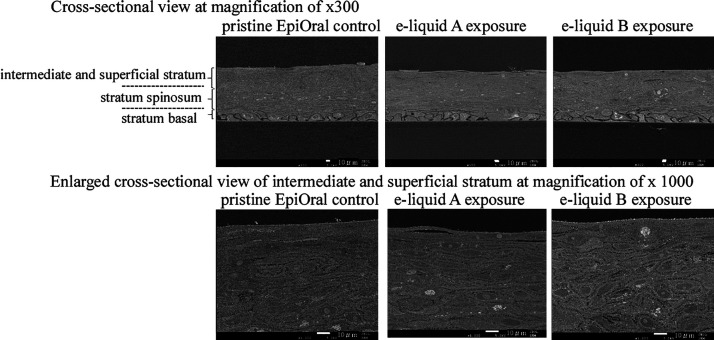
SEM images of the pristine tissue and the tissues after e-liquid A and e-liquid B exposure.

In order to understand the state of the tissues apart from their viability, the tissue morphology was observed by the SEM. The SEM images of the pristine tissue, and the tissues of e-liquid A and e-liquid B after the aerosol exposure are shown in Figure 3. The overall ultrastructural appearance of the three buccal epidermises was highly similar to that of a published report.^20^ All major epithelia including the stratum basal, stratum spinosum, intermediate stratum, and superficial stratum were present. The absence of significant differences of SEM images before and after the aerosol exposure revealed the absence of keratinization during the aerosol exposure and that the tissues maintained their state before the aerosol exposure. Also, the white grains in the SEM images were considered to be lipid droplets. These droplets were found in cultures of Epiderm (MatTek Corporation), which represents a highly differentiated, three-dimensional, cultured skin tissue equivalent.^27^ Thus, no morphological change occurs with the aerosol exposure, and a large number of gaps have been observed between cells, suggesting that absorption through this gap is likely to occur.

### 3.3 Tissue Absorption Studies

A preliminary test was performed to confirm over 90% recovery of PG, nicotine, and benzoic acid at the spike and recovery tests. The limit of detection (LOD) and limit of quantification (LOQ) were determined by using 3 times and 10 times the standard deviation of the mean weight, respectively, which was calculated from six replicates.^37,38^ The LOD and LOQ are 0.018 and 0.059 μg/mL for nicotine, 0.007 and 0.024 μg/mL for benzoic acid, and 0.4 and 1.5 μg/mL for PG. The average values of aerosol exposure of PG, nicotine, and benzoic acid were 293, 49, and 40 μg, respectively.

**Figure 4:**
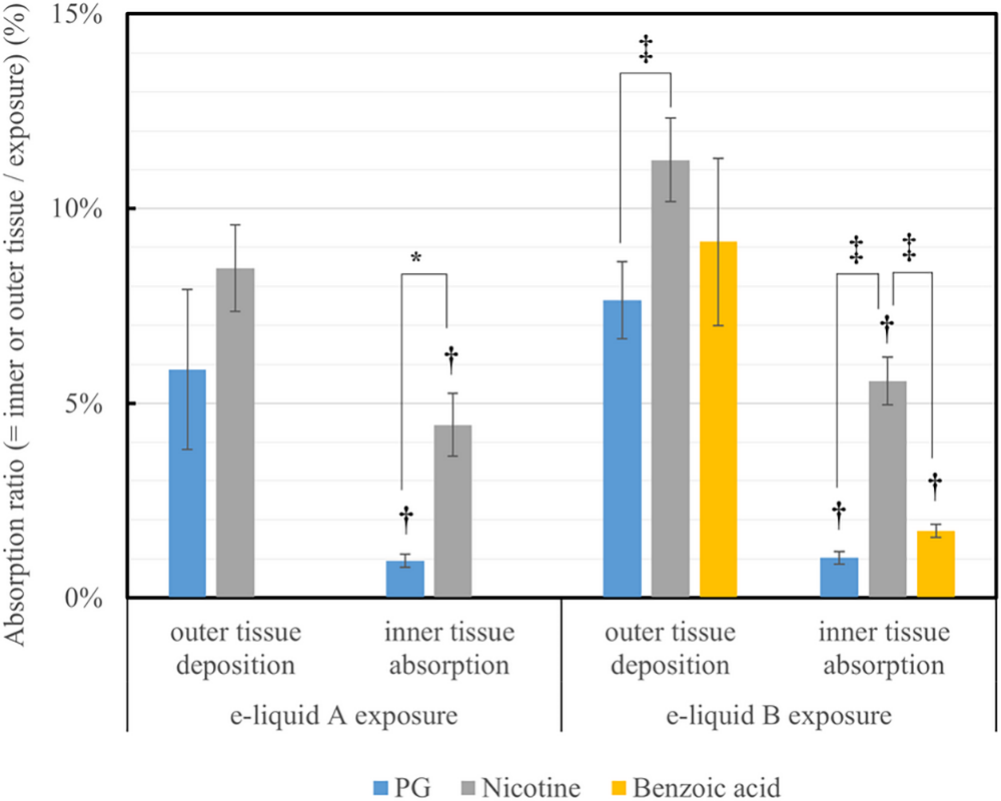
Absorption ratio of the value of inner tissue absorption and outer tissue deposition to the value of aerosol exposure, which was determined by quantifying solutions in the inserts filled only with water. *P < 0.05 (Welch’s t-test against the PG value). ^†^*P* < 0.05 (Welch’s t-test against corresponding outer tissue value). ^‡^*P* < 0.05 (one-way ANOVA followed by Tukey’s post hoc test).

**Figure 5:**
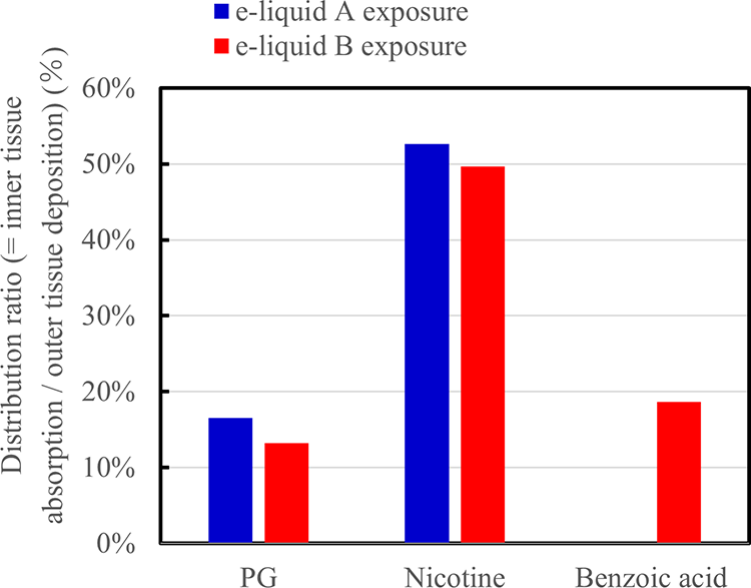
Distribution ratio of inner tissue absorption to outer tissue deposition after aerosol exposure.

The absorption ratio of the value of inner tissue absorption and outer tissue deposition to that of the aerosol exposure is shown in Figure 4. The distribution ratio of the value of the inner tissue absorption to that of the outer tissue deposition is shown in Figure 5. In Figure 4, the desorption ratio ranges from 6 to 11%, and it is worth noting that the desorption ratio is relatively low.

### 3.4 Absorption Selectivity

For the e-cigarette inhalation, approximately 20% of inhaled nicotine has been published to be absorbed in the buccal cavity, 75% in the respiratory tract, and 5% exhaled.^1^ Although the deposition ratio in our study is less than that in the published human data, these data show that most of the nicotine in the aerosol phase released from an e-cigarette does not adhere to the tissue and transfers instantly to the airway tissues. Unless the aerosol transfers to the airway tissues, the e-liquid compounds are eluted with the saliva after deposition on the epithelium and transferred partially to the inner tissue in parallel and partially to the GI tract accompanying multiple executions of swallowing. As shown in Figure 5, the distribution ratio of the value of the inner tissue absorption to that of the outer tissue deposition has selectivity and decreases in the order of nicotine ≫ benzoic acid > PG. Nicotine without the hydroxyl group has an increase in the absorption rate by approximately 5 times compared to water-soluble PG and benzoic acid with the hydroxyl group and has more lipophilic compounds than the others. This absorption selectivity demonstrates that the absorption depends on the affinity with the buccal epithelium rather than the passive diffusion via the gap as shown in SEM Observation. Additionally, the obtained distribution ratio indicated that, for nicotine, half transferred to the inner tissue via the oral epithelium and the other half transferred to the GI tract. On the other hand, for propylene glycol and benzoic acid, majority were eluted with the saliva and transferred to the GI tract.

Since nicotine is a dibasic nitrogen group with p*K*_a_’s of both 3.1 and 8.0 and a positively charged molecule in its protonated state such as in acidic environments, the charged molecules do not rapidly increase nicotine in the plasma across the oral cavity. Nicotine absorption studies have reported that the amount of absorption onto the oral epithelium increases with an increase in the amount of unprotonated nicotine in aqueous solution.^39−41^ Since the human mucous membrane remains in neutral conditions of pH and the unprotonated nicotine only exists in the epithelial surface, the absorption rate on the buccal epithelial surface would be assessed to be a rate limiting step of nicotine uptake in the plasma across the oral cavity. There is a slight scatter between the percentage of protonated nicotine in the aerosol and the estimation using the Henderson–Hasselbalch equation due to the effect of polyol (i.e., PG and G mixture).^42,43^ In this study, although the percentage of protonated nicotine of e-liquid B was relatively higher than that of acid-free e-liquid A based on the Henderson–Hasselbalch equation,^33^ the nicotine absorption ratio of the e-liquid B with the benzoic acid was almost the same as that of the acid-free e-liquid A, as shown in Figure 5. This suggested that the equilibrium of the protonated and unprotonated states or the dissociated state of ion and salt might change from the aqueous solution in the limitation of the proton concentration in the aerosol of polyol (i.e., PG and G mixture). As a result, the affinity between the buccal epithelium and nicotine in the aerosol droplets of polyol was different from that in the aqueous solution.

### 3.5 Absorption Mechanism

Many studies on the absorption of the buccal tissue appeared to discuss whether the majority of compounds pass across the paracellular and transcellular pathways.^14,44−46^ Since the tissue membrane consists of the hydrophobic compounds such as phospholipids, the lipophilic compounds can be absorbed via the transcellular pathway and hydrophilic compounds can be absorbed via the paracellular pathway.^44,46^ An enhancer having a high affinity with the tissue membrane or paracellular lipids has been studied to promote the tissue absorption.^44−47^ It has been pointed out that the exposure to ethanol or oleic acid may act as an absorption enhancer, possibly by causing a molecular rearrangement of the absorption barrier and the fluidization of the lipid layer in the outer portion of the epithelium.^48,49^

The absorption mechanism of the oral tissue was proposed to be attributed to a local and temporal dynamic topological deformation of the chemical structure caused by the fluctuations of lipid membrane structures, for example, a bicontinuous cubic phase transition with the change in the membrane surface curvature as shown in the absorption of protein-introduced domains with cationic charges.^14,50^ To understand the effect of the lipid structure on the absorption, we studied the structure and morphology change of the buccal tissues. As shown in Figure S1, the SAX/WAX analysis indicated that the structure of the EpiOral tissue did not change after the aerosol exposure and the liquid crystal structures of lipids such as phospholipids and glucosylceramide exist in the buccal epithelium. This indicated that the absorption mechanism in the buccal epithelium might play an important role in the affinity with the liquid crystal structure of phospholipids and glucosylceramide. However, the lipid ratio of phospholipids to glucosylceramides of the EpiOral tissue was less than that of the native buccal tissue, as mentioned above. The lipid structure of the non-keratinized epithelium has not yet been understood, and further interface scientific study is expected to focus on the surface adsorption of the non-keratinized epithelium. To understand the absorption via the paracellular pathway, a membrane of phospholipids and glucosylceramide having almost the same absorptive capacity as the human buccal epithelium and impacts of compounds on the structure or properties of this membrane will be investigated in the future.

### 3.6 Human Exposure Route

The absorption ratio of nicotine depends on the thickness and surface area of the organ, and it is slowed down in the order of the lower respiratory tract (lung) > the upper respiratory tract > the oral cavity.^2^ When the inhaled aerosol reaches the small airways and alveoli of the lung, nicotine is rapidly absorbed.^4^ The concentrations of nicotine in the plasma reached the maximum value immediately after cigarette smoking via the transpulmonary route as mentioned above.^4^ On the other hand, if nicotine retained and solved in the oral saliva is not absorbed from the oral epithelium, nicotine will flow into the GI tract.^2^ Nicotine is poorly absorbed from the stomach due to the acidity of gastric fluid^51^ but is well absorbed in the small intestine, which has a more basic condition and a large surface area.^52^ After absorption into the portal venous circulation, nicotine is metabolized by the liver before it reaches the systemic venous circulation.^41^ Although the absorption rate across the oral epithelium is faster than the absorption rate via the GI tract, the concentrations of nicotine in the plasma rise gradually, reaching the maximum value at approximately 30 min, and decline slowly over 2 h.^1,4^ Under our experimental conditions of aerosol exposure, majority of aerosol transfers to the airway tissues with less deposition on the oral epithelium. However, the findings of this study indicate that nicotine was absorbed to some extent across the oral mucosa when inhalable aerosols with acid were used, although it has been reported that nicotine absorption from the oral mucosa is relatively almost low when nicotine in the acid aqueous solution is used. Thus, some parameters of deposition and absorption as well as swallowing in the oral cavity were found to be closely related to the determination of the human exposure route.^1,2^ Furthermore, in order to understand the pharmacokinetics and physiological effects of the product on the user, it is necessary to understand the tissue-to-blood transfer.

## 4 Conclusions

In order to better understand the mechanisms of aerosol absorption via the buccal epithelium, an aerosol exposure test from an e-cigarette was performed using the EpiOral tissue model. The e-cigarette aerosol showed no decrease in the tissue viability and had no change in the cellular morphology and structure. It is worth noting that the absorption ratio of the value of inner tissue absorption and outer tissue deposition to that of the aerosol exposure is relatively low and shows that there is little deposition of aerosol on the surface of the oral epithelium based on this in vitro model. The distribution ratio of the value of the inner tissue absorption to that of the outer tissue deposition decreases in the order of nicotine ≫ benzoic acid > PG, and nicotine has an increase in the absorption rate by approximately 5 times compared with PG. This absorption selectivity depends on the affinity with the liquid crystal structure of the buccal epithelium rather than the passive diffusion via the gap as shown in SEM Observation. The nicotine absorption was almost the same regardless of the acid addition. This means that the affinity between the buccal epithelium and the aerosol droplets of polyol is different from that in the aqueous solution. Finally, the absorption across the oral cavity, respiratory tract, and GI tract was affected by the deposition and absorption and swallowing via the oral epithelium. Therefore, it is necessary to understand the absorption onto liquid crystal structures such as phospholipids and glucosylceramides, which have selective absorption on the oral epithelium and is closely related to the human exposure route.

## Supporting Information Available

SAXS/WAXS analysis of the EpiOral tissue after the aerosol exposure (PDF)
