# Supplementary material for: Oral Absorption across Organotypic Culture Models of the Human Buccal Epithelium after E-cigarette Aerosol Exposure
Source: ACS Omega. 2022 Dec 1;7(49):45574–81. doi: 10.1021/acsomega.2c06304 (PMC9753183; doi:10.1021/acsomega.2c06304)
Supplement: Supplementary file 1 — ao2c06304_si_001.pdf [file ao2c06304_si_001.pdf]

Supporting Information for

Oral absorption across organotypic culture models of the human buccal epithelium after e-cigarette aerosol exposure

*Masato Miyauchi † *, Shinkichi Ishikawa ‡ , Takeshi Kurachi ‡ , Kazutami Sakamoto§ and Hideki Sakai§*

† Tobacco Science Research Center, R&D Group, Japan Tobacco Inc., 6-2 Umegaoka, Aoba-ku, Yokohama, Kanagawa, 227-8512, Japan.

‡ Scientific Product Assessment Center, R&D Group, Japan Tobacco Inc., 6-2 Umegaoka, Aoba-ku, Yokohama, Kanagawa, 227-8512, Japan

§Department of Pure and Applied Chemistry, Faculty of Science and Technology, Tokyo University of Science, 2641 Yamazaki, Noda, Chiba 278-8510, Japan.

* Corresponding Authors: Masato Miyauchi, E-mail: masato.miyauchi@jt.com Tel: +81-80-1290-4329

SAXS/WAXS analysis of the EpiOral tissue after aerosol exposure

Experimental Section

In order to confirm no structure changes of the EpiOral tissue after the aerosol exposure, a small angle X-ray scattering (SAXS) and a wide angle X-ray scattering (WAXS) measurement were performed with a small angle X-ray scattering (Nano-viewer, Rigaku, Tokyo ,Japan) with Cu Kαradiation at 40kV and 30mA. The apparatus was incorporated with a detector of HyPix-3000 high-energy-resolution 2D detector. Three pieces of tissues, with approximately 3 mg in weight of dried sample, were put in the measurement holder while being sandwiched between polyester film. All samples were measured for a period of 15 min at the room temperature. Before the SAXS/WAXS measurements, they were kept refrigerated to suppress cellular structural changes. Three polycarbonate membranes used for the EpiOral tissue were overlaid to prepare a blank. The intensity of scattered two-dimensional X-ray was converted to one-dimensional intensity by circular averaging round the origin results in the x-ray scattering intensity.

Results and discussion

For the pristine EpiOral tissue, and the EpiOral tissues after the aerosol exposure of e-liquid A and e-liquid B, the SAXS and WAXS patterns isolated from the one-dimensional

intensity of each EpiOral tissue with polycarbonate substrate (Figure S1). Although the SAXS image around Q of 0.3nm^{-1} was difficult to isolate the SAXS pattern from the polycarbonate membrane with a high anisotropy, the SAXS and WAXS patterns of three tissues over q of 0.3 nm^{-1} was the same. As shown in Figure S1, the SAXS pattern revealed the presence of the peak at 11.8nm (corresponds to q of 0.53 nm^{-1}), which does not represent higher order SAXS patterns, and the WAXS patterns revealed the presence of the broad peak at 0.3nm (corresponds to q of 20 nm^{-1}). The lipophilic membrane, which depends on the absorption of the human buccal epithelium, consists mainly of the phospholipids (i.e, sphingomyelin, phosphatidylcholine, phosphatidylserine, phosphatidylinositol, phosphatidylethanolamine) and glucosylceramide.[S1] The SAXS/WAXS pattern analysis indicates the existence of liquid crystal structures of lipid membrane such as phospholipids and glucosylceramide in the buccal epithelium[S2] and the structure of the EpiOral tissue did not change after the aerosol exposure. That is, the lipid membrane is more densely arranged due to the attractive interaction between phospholipid and glucosylceramide, and the structure and properties of its lipid membrane exerts an influence on the absorption onto the buccal epithelium. Therefore, the absorption mechanism in the buccal epithelium was suggested to play an important role in the absorption into the liquid crystal structure of lipid.

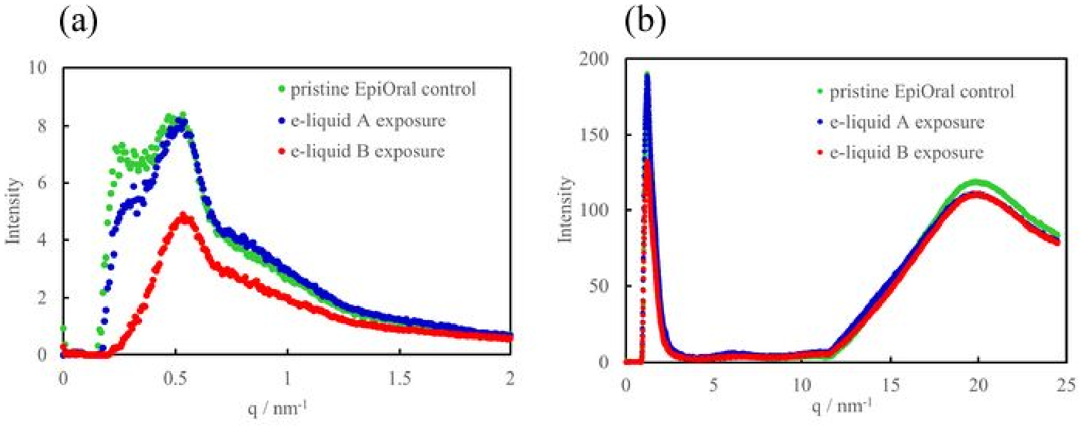

Figure S1. (a) SAXS pattern and (b) WAXS pattern of the pristine EpiOral tissue, and the EpiOral tissues after the aerosol exposure of e-liquid A and e-liquid B.

REFERENCES

S1 Law, S.; Wertz, P. W.; Swartzendruber, D. C.; Squier, C. A. Regional variation in content, composition and organization of porcine epithelial barrier lipids revealed by thin-layer chromatography and transmission electron microscopy. *Arch. Oral Biol.* **1995**, 40(12), 1085-1091. DOI: 10.1016/0003-9969(95)00091-7

S2 The society of cosmetic chemists of Japan, Encyclopedia of cosmetics, Maruzen Publishing Co., Ltd., Tokyo (Japan) 2013
